# Influencing factors and mechanism of tourists’ pro-environmental behavior – Empirical analysis of the CAC-MOA integration model

**DOI:** 10.3389/fpsyg.2022.1060404

**Published:** 2022-11-28

**Authors:** Haiyan Tang, Yong Ma, Jie Ren

**Affiliations:** ^1^School of Tourism Management, Hubei University, Wuhan, China; ^2^HuBei Tourism Development and Management Research Center, Wuhan, China; ^3^Department of Tourism Management, School of Business Administration, Zhongnan University of Economics and Law, Wuhan, China

**Keywords:** tourists’ pro-environmental behavior, the cognitive-affective-conative theory, the motivation-opportunity-ability theory, environmental self-identity, environmental commitment

## Abstract

Tourism has been perceived as an environmentally friendly industry for a long term, but the negative impact of tourist irresponsible behavior on the environment cannot be ignored. Tourists’ pro-environmental behavior is crucial to the destination’s sustainable development. Taking stimulus-organism-response theory as a framework, this study explores the factors and mechanisms influencing tourists’ pro-environmental behaviors by integrating cognitive-affective-conative (CAC) theory and motivation-opportunity-ability (MOA) theory. Through the PLS-SEM analysis with 548 questionnaires, the driving mechanism of tourists’ pro-environmental behavior was deeply explored. The results indicate that tourists’ environmental knowledge, participation motivation, and opportunity have significant positive effects on pro-environmental behavior, but the role of participation ability on tourists’ pro-environmental behavior is not confirmed. Tourists have significant environmental self-identity and environmental commitment, and these affections actively promote tourists’ pro-environmental behavior. Except for the participation ability, the other three factors, respectively, influence tourists’ environmental behavior through the mediating effects of environmental self-identity and environmental commitment. Given the limited literature integrating CAC and MOA theories within research on tourists’ pro-environmental behavior, these findings provide new perspectives for future research. This research enriches the literature on the factors influencing tourist pro-environmental behavior and also provides practical guidance for promoting tourists’ pro-environmental behavior.

## Introduction

Although tourism has been considered as a green industry, the burden of tourism development on the destinations’ ecological environment cannot be ignored (Danish and Wang, 2019; Luo et al., 2020). In addition to a large number of environmental problems caused by the development and management activities of the destination, the continuous growth of tourists has an increasingly negative impact on the environment (Juvan and Dolnicar, 2017; Zhang et al., 2019; Benner, 2020). Even if the intensity of tourism activities is low, it still hurts the ecological environment (Li et al., 2019b). Problems such as carbon emissions from transportation and accommodation (Lin et al., 2018; Betta et al., 2021), air pollution (Unger et al., 2016), increased solid waste (Hayati et al., 2020), and biodiversity destruction (Lin, 2022) created serious environmental problems. Tourists, as the main participants and key stakeholders in tourism, their irresponsible behaviors have been widely noticed by society. Tourists’ irresponsible travel behaviors (e.g., littering, landscape destruction, and ecosystem destruction) will bring enormous pressure on the environmentally sustainable development of the destination. The practices of tourists’ pro-environmental behavior can reduce damage to the environment and enhance the environmental quality of tourism destinations. Scholars have examined and confirmed that tourists’ pro-environmental behavior can contribute to the sustainable development of tourism destinations (Landon et al., 2018; Liu et al., 2019). To enhance people’s awareness of responsible travel, the government has enacted aggressively related laws and regulations to restrain tourists’ irresponsible behaviors. However, the external binding force has limited effects on promoting the positive behavior of tourists to a certain extent (Luo et al., 2020). Scholars have gradually realized that internal factors are driving more pro-environmental behavior of tourists. Thus, a large number of studies have extensively explored the influencing factors and contributing mechanisms on promoting tourists’ pro-environmental behavior (Han, 2015; Ateş, 2020).

Pro-environmental behaviors are those behaviors that tourists perform during tourism activities to reduce the negative impact on the destination environment and promote the sustainability of resource use (Halpenny, 2010; Lee et al., 2013). Currently, scholars have identified the driving factors of tourists’ pro-environmental behavior from different theoretical perspectives. From the rational perspective, the theory of rational behavior and the theory of planned behavior have a strong predictive effect on tourists’ behavior (Paco and Lavrador, 2017; Chwialkowska et al., 2020; Zebardast and Radaei, 2022). Integrating theoretical work on values and norm-activation processes, the value-belief-norm theory proposed by Stern et al. (1999) elucidates the driving mechanisms of individual pro-environmental behavior in terms of the activation of individual biosphere value, self-interest values, and altruistic values. As well, the sense of place theory provides theoretical contributions to identify factors influencing tourists’ pro-environmental behavior from a person-place affective perspective (Halpenny, 2010; Ramkissoon et al., 2012; Masterson et al., 2017). The stimulus-organism-response (SOR) theory has been widely applied in the explanation of pro-environmental behavior (Su and Swanson, 2017; Balaji et al., 2019; Kwon and Boger, 2020; Ye et al., 2020).

With further research, scholars have argued that cognitive and affective factors play an important driving role in tourists’ pro-environmental behavior (Baird et al., 2022). Among the existing research, few studies have explored the influencing factors that affect tourists’ pro-environmental behavior through motivation-opportunity-ability (MOA) theory and cognitive-affective-conative (CAC) theory conjointly. According to CAC theory, cognitive factors (e.g., knowledge and awareness) shape an individual’s attitudes and feelings, which in turn stimulate one’s behavior (Lavidge and Steiner, 1961). In addition, The MOA theory presents a valid theoretical basis for explaining an individual’s behavioral response in terms of motivation, opportunity, and ability. An individual’s motivation stimulates behavior more significantly when he/she has the necessary ability and is supported by external environmental opportunity. Since the MOA theory has strong explanatory power for consumer behavior and has been introduced into the research of tourists’ behavior. However, there is still lacking studies that apply MOA theory to explain tourists’ pro-environmental behavior (Lepoša, 2017; Pham et al., 2019). Combining the CAC theory and MOA theory, environmental knowledge plays an important role in driving tourists’ pro-environmental behavior (Ienna et al., 2022). Participation motivation and ability can stimulate tourists’ pro-environmental behavioral intentions. Additionally, the participation opportunity provided by the external environment drives tourists’ pro-environmental behavior. Environmental self-identity and environmental commitment play important roles in enhancing visitors’ pro-environmental behavior as individual psychological involvement conveys individuals’ environmental affective attitudes and concerns.

The tourists’ inappropriate behaviors not only bring negative effects to tourist attractions but also hinder the sustainable development of tourist destinations. Currently, it is an important issue to guide and enhance tourists’ pro-environmental behavior in academia. Assessing the factors influencing tourists’ pro-environmental behavior is significant to improve tourism sustainability (Zhu et al., 2022). Therefore, this research constructs mechanisms of tourists’ pro-environmental behavior based on the SOR theory, combined with the CAC theory and MOA theory. Based on the theoretical discussion, this research seeks to fill the research gaps by focusing on the influencing factors and paths of tourists’ pro-environmental behavior. Specifically, this research uses empirical analysis to assess the relationship between environmental knowledge, participation motivation, participation opportunity, participation ability, and tourists’ pro-environmental behavior through the mediating role of environmental self-identity and environmental commitment. This research will provide significant theoretical and practical references to improve tourists’ pro-environmental behavior in destination sustainable development and management practices.

## Theoretical framework and literature review

### The SOR theoretical framework

The Stimulus-Organism-Response (SOR) theory proposed by environmental psychologists Mehrabian and Russell (1974) includes three important components: stimulus (S), organism (O), and response (R). The SOR framework suggests that an individual stimulated by an internal or external factor (S) will change his or her psychology and emotions (O), and take corresponding behaviors (R) in response to it. Stimulus (S) is the determinant that influences a behavioral decision which may originate from the individual’s internal and external environment (Su and Swanson, 2017; Wang et al., 2021a). The elements that represent external stimuli include the overall atmosphere (e.g., consumer atmosphere and work environment support), visual (e.g., pictures and colors), and auditory (e.g., videos and music; Lorenzo-Romero et al., 2016). Moreover, the individual’s skills (Rose et al., 2012), risk perception (Wang et al., 2022), and participation motivation (Kamboj et al., 2018) could likewise influence the individual’s emotional state. Organism (O) refers to the psychological or emotional state that an individual develops in response to a stimulus which reflects the individual’s internal processing of the stimulus (Lee et al., 2011). Internal processing includes sensory and thinking activities, which can be specifically expressed as individual attitudes and emotions (Lorenzo-Romero et al., 2016). The term response relates to the final result and behavior triggered by the stimulus, which includes approach or avoidance behavior (Manthiou et al., 2016). Approach behavior is a positive response to a stimulus (e.g., the desire to look around, explore, stay, work, or positive communications), whereas avoidance behavior is the opposite (Mehrabian and Russell, 1974; Ul Islam and Rahman, 2017).

The SOR paradigm has well explained the emotional changes and behavioral characteristics of individuals and has been widely validated in many research domains. The framework has been extended to many other research fields, like online behavior (Rose et al., 2012), consumer behavior (Kwon and Boger, 2020), and organizational behavior (Attiq et al., 2017). The SOR theory has also been frequently applied in tourism research. In virtual tourism, the SOR theory has provided a valid theoretical contribution to clarify the relationship between stimuli (i.e., authentic experience and cognitive), organisms (i.e., affective responses and attachment), and visit intention (Kim et al., 2018). Based on the SOR theory, Chen et al. (2021) found that destination attributes have a critical function in honeymoon travel, which positively affected tourists’ emotions and satisfaction, and in turn drove their behaviors (i.e., revisit intention and word-of-mouth willingness). In nature-based tourism, natural soundscape positively stimulates visitors’ emotional arousal and emotional pleasure, which in turn promotes visitors’ actual approach and behavior (Jiang, 2020). Meanwhile, Wang et al. (2022) explored the influencing factors in medical tourism using the SOR theory and found that risk perception (stimulus) has a significant impact on tourists’ pandemic prevention attitude and decision-making (organism). In the current research, the SOR theory has been considered as an effective theory to explain individuals’ pro-environmental activities, especially green consumption behaviors (Balaji et al., 2019; Ye et al., 2020), employees’ energy-saving behaviors (Tang et al., 2019), and pro-environmental intentions (Kwon and Boger, 2020).

### The CAC theory

The theoretical framework in the present study mainly derived from the trilogy of mind, namely Cognitive-Affective-Conative (CAC) theory (Lavidge and Steiner, 1961; Hilgard, 1980). The core idea of the CAC theory is that an individual’s affective preference toward things is generated from cognitive evaluation and eventually shapes an individual’s attitude and behavioral decision (Oliver, 1999). It is shown that cognition will lead to individual mood changes. While cognition is often related to an individual’s perception and understanding of things, including perception, value, belief, and knowledge (Agapito et al., 2012). Affection represents associated feelings and attitudes which may be positive or negative (Kim et al., 2013; Huang et al., 2018). Conation is the expression of an individual’s intention, expressed as possible or actual behaviors, such as purchase intention (Park et al., 2008). In view of its effectiveness in explaining attitudes and behaviors, the CAC theory has been widely used in research on internet users’ sharing behavior (Hsiao, 2020) and consumer behavior (Lim and Kim, 2020). In tourism research, some researchers have applied the CAC theory to examine tourists’ loyalty, and others have used this theory to explain tourists’ environmentally responsible behavior (Ahn and Back, 2017; Liu et al., 2022).

The CAC theory plays a significant role in explaining an individual’s behavioral process. Therefore, the current study will take it as a theoretical basis to further explore the formative mechanisms of tourists’ pro-environmental behavior. Specifically, tourists’ environmental-related knowledge will influence their initial judgments when carrying out activities in tourist destinations, from which they will generate certain emotions and feelings. This research will focus on exploring the environmental self-identity and environmental commitment of tourists. The environmental self-identity and environmental commitment generated by tourists will drive their actual pro-environmental behavior. In other words, according to the CAC theory, tourist environmental knowledge (cognitive stage) will stimulate the environmental self-identity and environmental commitment (affective stage), which in turn influence the pro-environmental behavior (conative stage).

#### Cognitive: Environmental knowledge

Environmental knowledge is defined as general knowledge of facts, concepts, and solutions to environmental issues, and reflects the degree of understanding and concern for the relationship between humans and nature (Fryxell and Lo, 2003). Specifically, environmental knowledge is presented as individuals’ environmental literacy. Public environmental awareness and environmental knowledge have increased as a consequence of severe environmental problems globally, such as ecological damage, environmental pollution, and ecological disorders arising from tourism activities. Environmental knowledge has enhanced people’s awareness of environmental issues and promoted positive environmental choice (Abdullah et al., 2019). Amoah and Addoah (2020) investigated the relationship between environmental knowledge and family pro-environmental behavior. The results showed a positive relationship between environmental knowledge and pro-environmental behavior, suggesting that families with higher levels of environmental knowledge were more proactive about engaging in environmentally friendly activities. However, a lack of environmental knowledge can inhibit people from understanding environmental mitigation policies and implementing pro-environmental behavior (Bord et al., 2016; Unal et al., 2018). Tourists would often disregard their contributions to reducing negative environmental effects, without being acknowledged the specific environmental issues in the tourist destination and the ways to effectively mitigate them (Kim and Stepchenkova, 2019). Scholars have emphasized that environmental knowledge plays a significant role in shaping individuals’ values (Wang et al., 2018), attitudes (Ahmad and Thyagaraj, 2015), and behavioral intentions (Saari et al., 2021). For example, Fenitra et al. (2021) designed a theory to verify the relationship between environmental knowledge and attitudes. The theory emphasized that tourists’ attitudes toward positive environmental behavior would increase with a higher level of environmental knowledge. In addition, other studies have confirmed that environmental knowledge is positively related to environmental attitude and this relationship has a strong effect on behavioral intentions (Liu et al., 2020). As environmental knowledge increased, employees showed stronger environmental attitudes and greater willingness to take green behaviors (Safari et al., 2018). Similarly, Onel and Mukherjee (2016) indicated that individual environmental knowledge accumulation increased environmental risk perception and willingness to pay for environmentally friendly products, which therefore led people to perform more environmentally friendly behaviors.

#### Affective: Environmental self-identity and environmental commitment

The person-environment relationship structure provides a fundament for understanding tourists’ pro-environmental behavior (Rahman and Reynolds, 2016). The pre-existing understanding of the person-environment relationship includes environmental self-identity, connection to nature (Han, 2021), and environmental commitment (Davis et al., 2011). In this research, the role of environmental self-identity and environmental commitment in the mechanisms of tourists’ pro-environmental behavior formation will be taken into consideration.

Self-identity is a label that an individual uses to describe oneself (Cook et al., 2002). In other words, it defines “who I am” and emphasizes how the individual considers oneself. Thus, environmental self-identity can be defined as the extent to which one perceives oneself as an environmentally friendly person (van der Werff et al., 2013b). Having a strong environmental self-identity makes an individual more likely to recognize himself or herself as an environmentally friendly person and perform environmental actions according to the norms of those who are environmentally friendly. This means that environmental self-identity will promote environmental behavior when the self-concept sense is consistent with the connotation of the desired behavior. It should be noted that there is a conceptual difference between environmental identity and environmental self-identity. Environmental identity emphasizes the individual’s sense of belonging in the natural environment, (i.e., whether a person perceives himself or herself as part of the natural environment; Brügger et al., 2011). The latter refers to the level to which an individual regards acting with environmentalism as an important component of himself or herself (van der Werff et al., 2013b; Qasim et al., 2019). The role of environmental self-identity in predicting environmental behavioral intentions has been widely validated (Whitmarsh and O'Neill, 2010; Van der Werff et al., 2013a; Gil-Giménez et al., 2021; van der Werff et al., 2021). For example, individuals with a strong sense of environmental self-identity are more likely to consume organic food (Qasim et al., 2019), engage in recycling and environmental activities (Balunde et al., 2019), and have a greater willingness to consume energy efficiently and purchase sustainable products (van der Werff et al., 2013b).

Commitment is a widely researched topic in business operations and organizational management (Graci and Dodds, 2008; Rahman and Reynolds, 2016; Iftikhar et al., 2021). It plays an important role in enhancing corporate green innovation and performance and improving employees’ environmental behavior (Liu et al., 2012; Somjai et al., 2020). Commitment demonstrates the individual’s psychological state (Meyer and Allen, 1991) and is the internalization and identification of organizational goals (Cohen, 2007; Klein et al., 2014). Raineri and Paillé (2015) argued that beliefs and attitudes (e.g., environmental concern) related to the environment may not necessarily translate into actual behavior, whereas commitment is a strong facilitator of actual behavior. Since individual commitment is achieved by going over self-interest (Meyer and Herscovitch, 2001), which can provide a directivity of action for the individual or group (Davis et al., 2009). From the corporate perspective, environmental commitment is the process by which companies consider their environmental responsibilities and possible environmental problems in their business activities to minimize environmental damage (Hirunyawipada and Xiong, 2018). From the individual perspective (e.g., organization employees and consumers), environmental commitment represents the individual’s affective attachments, identification, and participation in environmental behavior (Cantor et al., 2012). Furthermore, environmental commitment describes the individual’s mind or psychological state, which expresses one’ s psychological attachment and responsibility to environmental issues (Afsar and Umrani, 2019). Internal (i.e., motivation and beliefs) and external (i.e., corporate social responsibility) factors influence environmental commitment, which in turn has a significant effect on individual pro-environmental behavior (Raineri and Paillé, 2015; Yusliza et al., 2020).

#### Conative: Tourists’ pro-environmental behavior

Realizing that tourism activities have many negative impacts on the ecological environment, a growing number of studies have focused on tourists’ behavior. As important stakeholders, tourists’ pro-environmental behavior can have a significant impact on destinations’ environmentally sustainable development. Pro-environmental behavior is the action of an individual to consciously reduce the negative impact on the environment (Kollmuss and Agyeman, 2010), such as recycling (Huber et al., 2018), green office (Tian and Robertson, 2017), energy consumption reduction (Liobikienė and Poškus, 2019), and other specific behaviors. The term pro-environmental behavior is interchangeably used with other terms such as environmentally responsible behavior, environmentally friendly behavior, green behavior, and eco-friendly behavior (Kiatkawsin and Han, 2017).

In tourism research, tourists’ pro-environmental behavior refers to the behavior of individuals or groups that promote the sustainable use of natural resources (Halpenny, 2010). Attribution theory argues that tourists’ pro-environmental behavior is influenced and interfered with by multiple objective and subjective factors. As for external drivers, environmental quality (Wu and Geng, 2020), incentives (Hu et al., 2018), and destination image (Zhang et al., 2020) are considered as antecedent variables that influence tourist pro-environmental behavior. In addition, internal factors might be cognitive and affective factors, such as risk perception (Yoon et al., 2021), environmental knowledge (Abdullah et al., 2019), place attachment (Ramkissoon et al., 2013b), values (Kim and Stepchenkova, 2019), and satisfaction (Ramkissoon et al., 2013a). In the CAC theory, pro-environmental behavior is an important content in tourists’ conative stage.

### The MOA theory

The Motivation-Opportunity-Ability (MOA) theory was proposed by MacInnis and Jaworski (1989) in their study on advertising information processing. According to the theory, individual attitude or behavior is formed by motivation (consumers’ desire or interest in processing brand information in an advertisement), opportunity (consumers’ attention to the brand in the advertisement), and ability (consumers’ ability to understand the brand information in the advertisement). It means that individual behavior intentions are more likely to be triggered by individual motivation (i.e., whether they want to do it or not), opportunity (i.e., whether they support it or not), and ability (i.e., whether they are in a condition to do it or not). The MOA theory has been widely applied in the research fields of human resource management and marketing (Van Waeyenberg and Decramer, 2018; Kellner et al., 2019; Sibian and Ispas, 2021).

In recent studies, scholars have extended the MOA theory to examine environmental behaviors. For example, Li et al. (2019a) applied the MOA theory to study the factors influencing energy-saving behavior in the workplace and found that opportunity had the greatest impact on employees’ energy-saving office behavior, followed by motivation and ability. In an empirical study, Pham et al. (2019) found that performance management (M), employee involvement (O), and training (A) could enhance the green behavior of hotel employees. The MOA theory has been applied to explain consumption behaviors in tourism research field. In leisure tourism activities, Lepoša (2017) explained the reasons for the conflict between the two roles of consumers and environmental citizens based on the integration of the consumer value theory and MOA theory. Given that the MOA theory is an appropriate approach to explain tourists’ behaviors, this research will incorporate the MOA theory into the research framework to explore the mechanism of tourists’ pro-environmental behavior from the aspects of individual motivation, opportunity, and ability.

## Hypotheses development and research model

Based on the above discussion of relevant literature, the theoretical framework of the present study extended the SOR theory by incorporating the MOA theory and the CAC theory. According to the framework, three key hypotheses have been made: (1) tourists are stimulated by multiple factors to produce psychological changes in the tourism environment, which in turn cause them to perform pro-environmental behavior; (2) tourists’ environmental knowledge (cognition) will inspire environment-related self-identity and commitment (affection), which in turn drives tourists’ pro-environmental behavior (conation); (3) participation motivation and participation ability factors are intrinsic factors that drive individual behavior, and participation opportunity is an external environmental factor that influences individual behavioral intention. To sum up, participation motivation, participation opportunity, participation ability, and environmental knowledge are considered as Stimulus (S) in the research framework, environmental self-identity and environmental commitment represent organism (O), and tourists’ pro-environmental behavior is considered as the response (R).

### Hypotheses based on the CAC theory

Some studies have confirmed the impact of certain knowledge on identity and commitment, such as the positive impact of food safety knowledge on food safety commitment (Taha et al., 2020). However, none of the existing studies have directly verified the relationship between environmental knowledge and environmental self-identity, and environmental commitment. Environmental knowledge describes the individual’s level of cognition about environmental issues and solutions. Environmental self-identity is a measure of an individual’s pro-environmental level, while tourists need to describe their pro-environmental level not only from the practical action consideration but also from the assessment of their environmental knowledge. Environmental commitment expresses the individual’s sense of responsibility for environmental issues, and it is impossible to practice environmental commitment actively without the guidance of scientific environmental knowledge. Accordingly, we argue that environmental knowledge is correlated with self-identity and environmental commitment. According to the CAC theory, affective responses depend on cognition. Cognitive factors such as awareness (Talwar et al., 2021) and perception (He et al., 2018) are antecedents of environmental self-identity and environmental commitment. Furthermore, Agnew et al. (1998) concluded that cognition and commitments were in a mutually influential relationship. We can assume that tourists’ level of environmental knowledge positively influences environmental self-identity and environmental commitment. That is the higher level of tourists’ environmental knowledge, the higher level of environmental self-identity and environmental commitment. Therefore, this research proposed the following hypotheses:

*H1*: Tourists' environmental knowledge positively influences environmental self-identity.

*H2*: Tourists' environmental knowledge positively influences environmental commitment.

Self-identity is thought to be highly related to environmental behavior. It has been suggested that environmental self-identity predicts behavioral consistency. For example, green self-identity is related to organic food consumption behavior (Carfora et al., 2019), sustainable self-identity is related to sustainable purchase behavior (Chen, 2020), and recycling self-identity is related to recycling behavior (Trudel et al., 2016). However, the generalized term of environmental self-identity is more widely used to predict environmentally friendly behaviors, including frugal behavior (Gil-Giménez et al., 2021), pro-environmental behavior (van der Werff et al., 2014), and energy consumption (Emmerich et al., 2020).

Individuals who make environmental commitments are more likely to practice their sense of environmental responsibility through actual actions. With high levels of environmental commitment, people are more likely to increase their pro-environmental behavior. Considering the role of environmental commitment in shaping behavior, it has also received considerable attention in tourism research (Sahabuddin et al., 2021; He et al., 2022). For example, individuals with different levels of environmental commitment during holiday travel differed in their choice of transportation mode for travel. Hergesell (2017) found that the higher the level of environmental commitment, the more environmentally conscious group preferred trains to cars. In addition, the environmental commitment of residents in tourist destinations promotes environmentally friendly behavior (Wang et al., 2021a). Based on the above findings, this study hypothesized that the arousal of environmental self-identity and environmental commitment in the tourism context would promote tourists’ pro-environmental behavior. Therefore, this research proposed the following research hypotheses:

*H3*: Environmental self-identity positively influences tourists' pro-environmental behavior.

*H4*: Environmental commitment positively influences tourists' pro-environmental behavior.

Environmental knowledge not only represents an individual’s level of awareness on relevant environmental issues, but also characterizes the degree that he or she cares about ecology. Environmental knowledge can increase people’s awareness of environmental issues, which in turn increases pro-environmental behavior. It has been shown in several empirical studies that environmental knowledge plays an important role in predicting individual behavioral intentions (Vicente-Molina et al., 2013; Liu et al., 2020). In the tourism research context, the more environmental knowledge tourists have, the more they care about the environmental issues in tourist destinations. With the development of environmental education and communication media, people’s environmental knowledge generally increases. As a result, the possibility of implementing pro-environmental behavior has been improved. With the wide use of social media, Han et al. (2017) found that pro-environmental knowledge in user-generated content played an important role in increasing tourists’ participation in pro-environmental tourism activities. Therefore, this research proposed the following research hypothesis:

*H5*: Environmental knowledge positively influences tourists' pro-environmental behavior.

### Hypotheses based on the MOA theory

In the original MOA theory, MacInnis and Jaworski (1989) proposed that motivation is related to an individual’s desires, which include interest, willingness, and readiness. Specifically, motivation describes the reasons for individual behavioral tendencies. Environmental self-identity reflects the individual’s psychological involvement in the person-environment relationship, and commitment can be understood as a motivational transformation process (Su et al., 2019). Environment-related participation motivations, such as promoting destination sustainability, will connect tourists to the sustainability of the destination and promote their pro-environmental self-identity. Accordingly, we argue that environmental self-identity and motivation are closely related, and participation motivation can strengthen the tourists’ environmental self-identity. While commitment is an individual’s sense of responsibility for environmental protection, personal beliefs and motivation directly affect their commitment. Based on motivation theory, scholars have found that motivation can increase the residents’ pro-environmental identity and pro-environmental commitment level in tourism destinations (Wang et al., 2019). Based on the above analysis, this research proposed the following hypotheses:

*H6*: Participation motivation positively influences environmental self-identity.

*H7*: Participation motivation positively influences environmental commitment.

Motivation is an important antecedent variable in explaining individual behavioral characteristics. The role of motivation in predicting individual attitudes and behavioral intentions has been widely confirmed. Regarding the influence of motivation on pro-environmental behavior, Tabernero and Hernandez (2012) empirically analyzed self-efficacy, internal motivation, and external motivation on individual pro-environmental behavior. The results indicate that internal motivation moderated the effect of self-efficacy on pro-environmental behavior. Based on classical motivation theory, Geng et al. (2017) studied the influence of two types of motivation on residents’ green travel, including pro-environmental motivation and self-interest motivation. These studies suggest that the tourists’ pro-environmental behavior is influenced by individual motivation. Therefore, this research proposed the following research hypothesis:

*H8*: Participation motivation positively influences tourists' pro-environmental behavior.

According to MacInnis and Jaworski (1989), opportunity refers to favorable conditions, including organizational support and interpersonal relationships. In this research, participation opportunity refers to the factors that are favorable for tourists to carry out tourism activities and perform pro-environmental behavior, mainly including the policies, channels, or facilities support provided by the government and tourism destinations. The external environment provides the opportunity for people to create conditions for specific participatory behaviors. For example, the establishment of communication channels and increased cooperation with tourism destinations can provide opportunities for tourists to make heritage tourism and promote heritage conservation behaviors (Aas et al., 2005). In organizational management, the company’s environmental policy support affects the level of environmental commitment, and there is a moderating effect of environmental commitment in the influence of the company’s environmental policy on environmental citizenship behavior (Raineri and Paillé, 2015). With relevant policies and environmental support from the tourism destinations and governments, tourists will have a stronger sense of environmental self-identity which will also trigger a higher level of environmental commitment. Therefore, this research proposed the following research hypotheses:

*H9*: Participation opportunity has a positive impact on tourists' environmental self-identity.

*H10*: Participation opportunity has a positive influence on tourists' environmental commitment.

*H11*: Participation opportunity has a positive impact on tourists' pro-environmental behavior.

According to the original concept of the theory, ability refers to consumers’ ability to understand advertising messages (MacInnis and Jaworski, 1989), which generally includes skills and resources (Lepoša, 2017). In energy consumption behavior, the ability is also considered as the interpretation, understanding, and reasoning of information about energy use (Li et al., 2019a). In the tourism context, participation ability includes factors like awareness, experience, skills, accessibility to information and financial resources. Additionally, sufficient time is one of the necessary conditions to carry out tourism activities. Therefore, in this research, participation ability includes time, financial resources, skills, and experience. The validity of MOA theory in predicting and explaining behavior was tested. Based on the MOA theory, Sibian and Ispas (2021) found that hotel employees’ insufficient ability in environmental awareness, concern, and skills had an inhibitory effect on employees’ green behavior. Ability (e.g., experience) related to the practice of pro-environmental tourism activities helps tourists to better understand the person-environment relationship, which is beneficial in promoting pro-environmental behavior. The stronger the participation ability, the higher the level of environmental self-identity and environmental commitment of the tourists, and the better they can enhance pro-environmental behavior. Therefore, this research proposed the following research hypotheses:

*H12*: Participation ability positively influences tourists' environmental self-identity.

*H13*: Participation ability positively influences tourists' environmental commitment.

*H14*: Participation ability positively influences tourists' pro-environmental behavior.

In addition, the research also considered the possible mediating role of environmental self-identity and environmental commitment separately. We proposed the following hypotheses:

*H15*: Environmental self-identity (a)/environmental commitment (b) mediates the effect of environmental knowledge on tourists' pro-environmental behavior.

*H16*: Environmental self-identity (a)/environmental commitment (b) mediates the effect of participation motivation on tourists' pro-environmental behavior.

*H17*: Environmental self-identity (a)/environmental commitment (b) mediates the effect of participation opportunity on tourists' pro-environmental behavior.

*H18*: Environmental self-identity (a)/environmental commitment (b) mediates the effect of participation ability on tourists' pro-environmental behavior.

**Figure 1:**
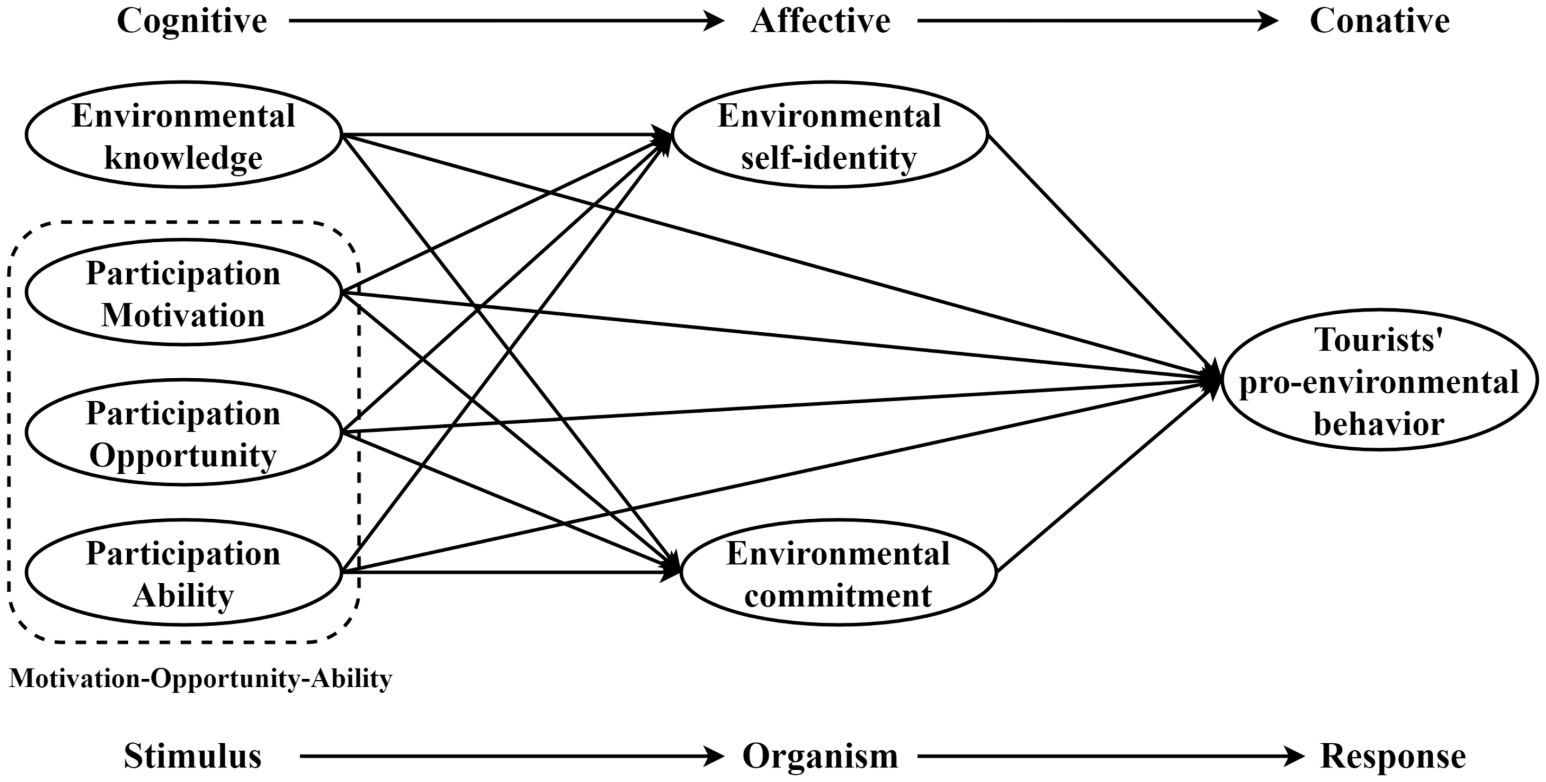
Model framework.

Based on the above discussion of the relationship between variables, this research exploratively proposed a research model for the influence mechanism of tourists’ pro-environmental behavior (Figure 1).

## Methodology

### Measurements

The measurement of variables involved in this research is derived from established scales in domestic and international studies with appropriate modifications, which ensures the validity and reliability of the research data. To avoid the semantic differences or cultural background differences affecting the quality of the questionnaire, the questionnaire was first translated independently by the research team members and formed into a primary Chinese version scale. Then, it is reviewed by three tourism experts and two PhDs in the relevant fields. Until the language was accurate and unambiguous, the research scale was determined. The four construct items of environmental knowledge are referenced from Kim and Stepchenkova (2019) study. The six items of participation motivation are based on the studies of Zhang et al. (2014), Kiatkawsin and Han (2017), and Wang et al. (2019). Three items of participation opportunity and four items of participation ability are referenced from the study of Liu and Shi (2021). The three items measuring environmental self-identity are referred to in the study of Werff et al. (2013). The four items for environmental commitment are taken from the studies of Davis et al. (2009), He et al. (2018), and Wang et al. (2019). Moreover, the five items to measure tourists’ pro-environmental behavior were drawn from the study by Chiu et al. (2014). The scale is all based on the Likert 5-point scale, which asks the questionnaire participants to score each question item from 1 (completely disagree) to 5 (completely agree).

### Sample and data collection

Due to the impact of COVID-19, this research collected questionnaire data from an online platform Wenjuanxing^1^. Since its launch in 2006, its users have covered more than 90% of universities and research institutes in China, with a total of over 13.23 billion responses. A screening question was designed to eliminate people who have not traveled in the last year. Before formally sending the research questionnaire, a pilot test has been conducted. The analysis of the 119 responses obtained showed that the Cronbach’s alpha values of the constructs ranged from 0.784 to 0.875. This indicated that the reliability of the questionnaire was good. Next, we formally started the research in August 2022. We eliminated questionnaires with unusual completion times and inconsistent answers. Finally, 548 valid questionnaires were obtained, with an effective rate of 91.3%.

Table 1 presents the basic characteristics of the survey respondents. Out of the 548 valid samples, 50.5% were male and 49.5% were female. The majority of the respondents were in the age ranges 30–39 (37.6%) and 40–49 (26.5%), 18.4% were 18–29, 12.4% were 50–59, and 4.9% aged over 60. Almost half of the respondents had a bachelor’s degree (45.6%) and 13.3% had a postgraduate degree.

**Table 1:** Main characteristics of survey respondents.

Variable	Group	Frequency (*N* = 548)	Percentage (%)
Gender	Male	277	50.5
	Female	271	49.5
Age	18–29	101	18.4
	30–39	206	37.6
	40–49	145	26.5
	50–59	68	12.4
	60 or above	27	4.9
Education	High school or below	225	41.1
	Bachelor degree	250	45.6
	Master’s degree or above	73	13.3

## Results

This study analyzed the influence mechanism of tourists’ pro-environmental behavior using partial least squares structural equation modeling (PLS-SEM). The empirical analysis was based on a two-step process. First, the measurement model was assessed to test the consistency reliability, convergent validity, and discriminant validity. Next, we assessed the structural model and tested the hypotheses.

### Assessment of the measurement model

Generally, consistency reliability, convergent validity, and discriminant validity are used to assess the measurement model. As shown in Table 2, the factor loadings of all construct terms ranged from 0.710 to 0.913, reaching a significant level. Meanwhile, the Composite Reliability (CR) and Cronbach’s Alpha (α) of the seven constructs were above 0.8, which was higher than the recommended value of 0.7, indicating that the constructs had good internal consistency. The convergent validity was assessed by calculating the value of the Average Variance Extracted (AVE). The results showed that the AVE values ranged from 0.644 to 0.805 (>0.5), therefore, the results supported the convergent validity. Additionally, Table 2 suggested that all indicators with the Variance Inflation Factor (VIF) values ranged from 1.598 to 3.066 (<3.3), so there was no covariance in this research (Schlittgen et al., 2015). To minimize the effect of common method variance (CMV) on the validity of the results generated by the tourists’ self-reported data, this research used Harman’s one-factor test for statistical testing. The first factor extracted by unrotated principal component analysis had a variance explained of 44.445%, which is below the critical value of 50% of the discriminant criterion (Podsakoff et al., 2003). Hence, there is no significant issue of common method variance in the data of this study.

**Table 2:** Items, constructs and measurement model evaluation results.

Constructs/indicators	Mean	St. dev.	Loading	VIF
**Environmental knowledge: α = 0.865; CR = 0.908; AVE = 0.713**				
I know what environmental problems human beings are facing (e.g., climate warming, air pollution, biodiversity reduction, etc.)	4.080	0.784	0.849	2.549
I know the problems and threats that tourism poses to the environment	4.090	0.774	0.896	3.066
I know the environmental slogans of tourist attractions	4.050	0.769	0.807	1.829
I know what behaviors can reduce the damage to the environment during tourism activities (such as carrying toiletries, not littering, etc.)	4.320	0.694	0.822	1.781
**Participation Motivation: α = 0.889; CR = 0.915; AVE = 0.644**				
My pro-environmental behaviors will be beneficial to protect the rights of other tourists or residents in the tourism destination during tourism activities	4.550	0.715	0.798	2.399
My pro-environmental behaviors will contribute to the environmental sustainability of the tourism destination	4.560	0.653	0.831	2.731
Taking active roles in protecting the destination environment benefits the whole ecosystem	4.630	0.625	0.810	2.566
Performing environmental protection behaviors in tourism activities can help me establish a good social impression	4.470	0.657	0.827	2.727
Consuming pro-environmental tourism products and services will show my social status	4.530	0.679	0.832	2.695
Consuming pro-environmental tourism products and services will enhance my sense of accomplishment	4.300	0.8	0.710	1.598
**Participation Opportunity: α = 0.879; CR = 0.925; AVE = 0.805**				
Government policies support and encourage tourists to perform pro-environmental tourism	4.400	0.699	0.888	2.354
Tourism destinations support pro-environmental tourism by actively designing and developing eco-tourism products	4.300	0.705	0.893	2.310
The government/destination offers channel support for tourists to develop pro-environmental tourism	4.320	0.692	0.910	2.715
**Participation Ability: α = 0.90; CR = 0.930; AVE = 0.770**				
I have enough time for pro-environmental tourism	3.770	0.914	0.863	2.370
I have the purchasing ability to undertake pro-environmental tourism	3.810	0.926	0.880	2.538
I hold knowledge related to pro-environmental tourism	3.860	0.854	0.870	2.515
I have the professional skills to perform pro-environmental tourism	3.640	0.921	0.896	3.018
**Environmental self-identity:α = 0.870; CR = 0.925; AVE = 0.805**				
Acting environmentally friendly is an important part of who I am	4.260	0.724	0.882	2.112
I am the type of person who acts environmentally friendly	4.200	0.719	0.913	2.828
I see myself as an environmentally friendly person	4.210	0.703	0.897	2.614
**Environmental commitment: α = 0.856; CR = 0.903; AVE = 0.699**				
Protecting tourism destination environment should be our priority, even if there is a conflict between economic growth and environmental protection	4.260	0.751	0.866	2.255
I am willing to buy a pro-environmental brand product, even if it costs more	3.960	0.792	0.800	1.777
I am willing to give up actions that are harmful to the natural environment	4.220	0.722	0.847	2.104
We should proactively take responsibility for environmental protection	4.450	0.659	0.829	1.943
**Tourists’ pro-environmental behavior: α = 0.866; CR = 0.904; AVE = 0.653**				
I will not leave litter when visiting a scenic spot	4.580	0.631	0.804	2.807
I will comply with the destination’s environmental guidelines	4.560	0.612	0.838	3.041
I will proactively persuade and prevent others from damaging the environment	4.020	0.864	0.712	1.701
I will remind my friends and relatives to avoid actions that damage the tourist environment	4.370	0.675	0.861	2.417
I will actively learn about environmental protection	4.270	0.741	0.819	2.128

α, Cronbach’s alpha; CR, composite reliability; AVE, average variance extracted; St. dev., standard deviation; VIF, variance inflation factor.

Next, we assessed the discriminant validity with the use of the Fornell-Larcker criterion (Fornell and Larcker, 1981). Table 3 showed that the square root of each construct’s AVE in this research was higher than the correlation coefficient between any two constructs, indicating that the constructs had favorable discriminant validity. However, due to the possibility of overestimation of AVE values by PLS, Henseler et al. (2014) suggested that the differential validity should be assessed again by the Heterotrait-Monotrait Ratio (HTMT). When all values of HTMT are below 0.9, the model is considered to have good discriminant validity. In Table 4, we observed that all values of HTMT were below 0.9, so the discriminant validity of the model was satisfactory.

**Table 3:** Fornell-Larcker discriminant validity criterion.

	EK	PM	PO	PA	ESI	EC	TPEB
Environmental knowledge (EK)	**0.844**						
Participation motivation (PM)	0.444**	**0.802**					
Participation opportunity (PO)	0.597**	0.672**	**0.897**				
Participation ability (PA)	0.629**	0.406**	0.553**	**0.877**			
Environmental self-identity (ESI)	0.700**	0.578**	0.682**	0.628**	**0.897**		
Environmental commitment (EC)	0.650**	0.585**	0.686**	0.591**	0.769**	**0.836**	
Tourists’ pro-environmental behavior (TPEB)	0.662**	0.609**	0.704**	0.544**	0.756**	0.749**	**0.808**

The diagonal value is the square root of AVE. ***Indicates *p* < 0.001, **indicates *p* < 0.01, and *indicates *p* < 0.05.

**Table 4:** HTMT discriminant validity criterion.

	EK	PM	PO	PA	ESI	EC	TPEB
Environmental knowledge (EK)							
Participation motivation (PM)	0.510						
Participation opportunity (PO)	0.687	0.758					
Participation ability (PA)	0.712	0.447	0.622				
Environmental self-identity (ESI)	0.805	0.650	0.775	0.706			
Environmental commitment (EC)	0.756	0.676	0.792	0.667	0.884		
Tourists’ pro-environmental behavior (TPEB)	0.760	0.700	0.803	0.591	0.858	0.866	

### Assessment of the structural model

We tested the structural model and the research hypotheses with the help of the PLS Algorithm, Blindfolding, and Bootstrapping method (5,000 repetitions of sampling). First, we evaluated the model fit by the standardized root mean square residual (SRMR) value. In this research, we obtained an SRMR value of 0.071 (less than 0.08), which indicated that the model specification was within the eligible threshold (Hu and Bentler, 1999; González-Rodríguez and Díaz-Fernández, 2020).

The values of the path coefficient (*β*), determination coefficient (*R*^2^), and the cross-validated redundancy measure (*Q*^2^) provide an important reference for assessing the structural model (Hair et al., 2014). *R*^2^ represents the effect of exogenous constructs on endogenous constructs and is used to assess the predictive accuracy of the model (Preziosi et al., 2019). The expected value of *R*^2^ ranges from 0 to 1, and the higher value indicates a stronger explanatory power. As a judgment criterion, the values of *R*^2^ higher than 0.25, 0.5, and 0.75 represent the predictive power as weak, moderate, and substantial, respectively (Hair et al., 2019). *Q*^2^ is used to assess the predictive relevance of the model, and the effect is significant when *Q*^2^ is larger than 0. As a judgment criterion, when the values of *Q*^2^ higher than 0, 0.25, and 0.50 depict small, medium, and large predictive relevance of the PLS-path model (Hair et al., 2019). In Table 5, the values of *Q*^2^ are all larger than 0.25, indicating that the path coefficients are significant (except for *H*14). The model explained 64.4% of environmental self-identity, 60.2% of environmental commitment variance and 68.8% of tourists’ pro-environmental behavior.

**Table 5:** Endogenous constructs assessment (*R*^2^ and *Q*^2^).

Construct	*R* ^2^	Adjusted *R*^2^	*Q* ^2^
Environmental self-identity (ESI)	0.644	0.641	0.509
Environmental commitment (EC)	0.602	0.599	0.414
Tourists’ pro-environmental behavior (TPEB)	0.686	0.682	0.440

Specifically, Table 6 reported the path coefficients, significance levels, and t statistics. The results indicated that environmental knowledge had a significant effect on environmental self-identity (*β* = 0.358, *p* < 0.001), environmental commitment (*β* = 0.285, *p* < 0.001), and tourists’ pro-environmental behavior (*β* = 0.176, *p* < 0.01), which supported *H*1, *H*2, *H*4 and *H*5. The effects of environmental self-identity and environmental commitment on tourists’ pro-environmental behavior (*β* = 0.260, *p* < 0.001; *β* = 0.265, *p* < 0.001) were positively significant. Hence, hypotheses *H*3 and *H*4 were supported. As well, participation motivation had a significant effect on environmental self-identity (*β* = 0.174, *p* < 0.001), environmental commitment (*β* = 0.203, *p* < 0.001), and tourists’ pro-environmental behavior (*β* = 0.130, *p* < 0.01), and hypotheses *H*6, *H*7 and *H*8 were supported. In addition, the effect of participation opportunity on environmental self-identity (*β* = 0.238, *p* < 0.001), environmental commitment (*β* = 0.290, *p* < 0.001) and tourists’ pro-environmental behavior (*β* = 0.178, *p* < 0.01) were positively significant, which supported hypotheses *H*9, *H*10 and *H*11. Lastly, participation ability significantly and positively affected environmental self-identity (*β* = 0.203, *p* < 0.001) and environmental commitment (*β* = 0.163, *p* < 0.001), and supported hypotheses *H*12 and *H*13. The effect of participation ability on tourists’ pro-environmental behavior (*β* = −0.056, *p* > 0.05) was not significant, hence hypothesis *H*14 was rejected.

**Table 6:** Model hypothesis statistics (bootstrapping).

Hypothesis	Path	*β*	*SE*	*T* statistics	LLCI	ULCI	Outcome
*H*1	EK → ESI	0.358***	0.047	7.684	0.268	0.442	√
*H*2	EK → EC	0.285***	0.045	6.294	0.193	0.371	√
*H*3	ESI → TPEB	0.260***	0.055	4.727	0.15	0.371	√
*H*4	EC → TPEB	0.265***	0.054	4.929	0.158	0.371	√
*H*5	EK → TPEB	0.176**	0.058	3.063	0.061	0.288	√
*H*6	PM → ESI	0.174***	0.049	3.520	0.088	0.274	√
*H*7	PM → EC	0.203***	0.058	3.486	0.103	0.328	√
*H*8	PM → TPEB	0.130**	0.047	2.766	0.048	0.23	√
*H*9	PO → ESI	0.238***	0.059	4.019	0.111	0.343	√
*H*10	PO → EC	0.290***	0.065	4.496	0.154	0.402	√
*H*11	PO → TPEB	0.178**	0.052	3.420	0.075	0.28	√
*H*12	PA → ESI	0.203***	0.041	4.904	0.127	0.285	√
*H*13	PA → EC	0.163***	0.046	3.545	0.077	0.257	√
*H*14	PA → TPEB	0.056	0.039	1.442	−0.129	0.02	×

EK, environmental knowledge; ESI, environmental self-identity; EC, environmental commitment; TPEB, tourists’ pro-environmental behavior; PM, participation motivation; PO, participation opportunity; PA, participation ability; *β*, path coefficient; SE, standard error; LLCI, lower limit confidence interval; ULCI, upper limit confidence interval. ***Indicates *p* < 0.001, ** indicates *p* < 0.01, *indicates *p* < 0.05.

Finally, we assessed the mediating role of stimulus factors (environmental self-identity and environmental commitment) between organism factors (environmental knowledge, participation motivation, participation opportunity, and participation ability) and tourists’ pro-environmental behavior. As shown in Table 7, the mediating effects of environmental self-identity and environmental commitment between environmental knowledge and tourists’ pro-environmental behavior were significant (*β* = 0.093, *p* < 0.001; *β* = 0.075, *p* < 0.001). Environmental self-identity and environmental commitment mediated the effect of participation motivation on tourists’ pro-environmental behavior (*β* = 0.045, *p* < 0.01; *β* = 0.054, *p* < 0.01). Meanwhile, the mediation effects of environmental self-identity and environmental commitment between participation opportunity and tourists’ pro-environmental behavior were confirmed (*β* = 0.062, *p* < 0.01; *β* = 0.077, *p* < 0.01). None of the bias-corrected lower limit (2.5%) and upper limit (97.5%) confidence intervals included zero. Hence, the mediation hypotheses for *H*15a, *H*16a, *H*17a, *H*15b, *H*16b, and *H*17b are well supported. While the mediating effects of environmental self-identity and environmental commitment between participation ability and tourists’ pro-environmental behavior were not confirmed in this research. Thus, *H*18a and *H*18b are not empirically supported.

**Table 7:** Summary of mediating effect test.

Hypothesis	Path	Total effect	Direct effect	Indirect effect	Mediating effect
*H*15a	EK → ESI → TPEB	0.345*** (0.246,0.437)	0.176*** (0.265,0.443)	0.093*** (0.05,0.142)	√
*H*16a	PM → ESI → TPEB	0.229*** (0.134,0.346)	0.13*** (0.045,0.229)	0.045** (0.02,0.081)	√
*H*17a	PO → ESI → TPEB	0.317*** (0.198,0.425)	0.178*** (0.075,0.28)	0.062** (0.024,0.106)	√
*H*18a	PA → ESI → TPEB	0.040 (−0.041,0.128)	0.056 (−0.129,0.02)	—	×
*H*15b	EK → EC → TPEB	0.345*** (0.246,0.437)	0.176*** (0.265,0.443)	0.075*** (0.04,0.116)	√
*H*16b	PM → EC → TPEB	0.229*** (0.134,0.346)	0.13*** (0.045,0.229)	0.054** (0.025,0.096)	√
*H*17b	PO → EC → TPEB	0.317*** (0.198,0.425)	0.178*** (0.075,0.28)	0.077** (0.034,0.125)	√
*H*18b	PA → EC → TPEB	0.040 (−0.041,0.128)	0.056 (−0.129,0.02)	—	×

EK, environmental knowledge; ESI, environmental self-identity; TPEB, tourists’ pro-environmental behavior; PM, participation motivation; PO, participation opportunity; PA, participation ability; EC, environmental commitment. ***Indicates *p* < 0.001, **indicates *p* < 0.01, *indicates *p* < 0.05; Values in parentheses represent the 97.5% confidence interval.

## Discussion

### Conclusion and discussion

Based on an empirical test, this study has broadened our understanding of tourists’ pro-environmental behavior by integrating the CAC theory and the MOA theory grounded in the SOR theory. Overall, this research assessed the role of stimulus and organismic factors in an attempt to reveal the driving mechanisms of pro-environmental behavior in tourists. Our research supports the effects of environmental self-identity and environmental commitment in the relationship between stimulus factors (environmental knowledge, participation motivation, and participation opportunity) and tourists’ pro-environmental behavior. Specifically, the following main findings are found in the research.

First, by investigating the direct effect of environmental knowledge on tourists’ pro-environmental behavior, the findings confirm that environmental knowledge positively affects tourists’ pro-environmental behavior, which supports previous research findings (Mostafa, 2007; Cheng and Wu, 2014; Abdullah et al., 2019). This research suggests that individuals with higher levels of environmental knowledge are more likely to protect the natural environment of their destination and perform pro-environmental behavior during tourism activities. In addition, in the MOA theory, participation motivation and opportunity also positively influence tourists’ pro-environmental behaviors. The effect of participation motivation on tourists’ pro-environmental behavior has been widely demonstrated (Clark et al., 2003; Faraz et al., 2021). Participation motivation is an important driving factor for tourists’ pro-environmental behavior, and the stronger the motivation for environmental concern or self-image enhancement, the more likely tourists are willing to support pro-environmental behavior. However, different from Li et al. (2019a) study, we observed that it was not significant when examining the effect of participation ability on the tourists’ pro-environmental behavior. Thus, the research rejected hypothesis *H*14. What is interesting to note is that in examining tourists’ social media engagement behavior, Leung and Bai (2013) found that ability was also not significantly influencing social media engagement behavior. This was because the respondents in their study already had a certain level of use of social media tools. In this research, the hypothesis of the relationship between ability and tourists’ pro-environmental behavior was not recognized. It may be that tourists are not required with complex skills or rich experience to perform pro-environmental behavior (e.g., to not litter or destroy vegetation) during tourism activities. Moreover, the tourism attractions’ management system also constrains the tourist to implement civilized tourism behaviors. These relationships also reflect the positive role of individual initiative and external opportunities in promoting tourists’ pro-environmental behavior. In addition, the findings support the positive effects of environmental knowledge, participation motivation, participation opportunity, and participation ability on tourists’ environmental self-identity and environmental commitment. Tourists’ level of environmental knowledge, participation motivation, participation opportunity, and participation ability all influence their thinking about the person-environment relationship and stimulate environmental self-identity and environmental commitment.

Secondly, the findings that environmental self-identity and environmental commitment positively influence tourists’ pro-environmental behavior are consistent with previous research findings (Dermody et al., 2015; Fanghella et al., 2019; Wang et al., 2021b). In the person-environment relationship, environmental self-identity and environmental commitment express a positive affective attachment, which is the individual or group’s emotional identification and belonging to the natural environment. Where environmental self-identity reflects how the tourists perceive “themselves,” the more profoundly they agree with their environmental identity, the more likely they are to support and perform pro-environmental behavior. Moreover, individuals with stronger environmental self-identity tend to perform pro-environmental behavior more often even in the absence of stimuli from external factors (Werff et al., 2013). Individuals’ previous pro-environmental behavior also enhances their environmental self-identity, which in turn enhances their pro-environmental behavior (Van der Werff et al., 2013a). Environmental commitment highlights the individuals’ sense of responsibility for the environment and requires sacrificing or going beyond the tourists’ benefits to achieve it. Commitment to protecting the environment and practicing pro-environmental behavior will positively lead to the pro-environmental behavior of tourists in their tourism activities.

Finally, we also find support for the research hypotheses that the relationship between stimulus factors (environmental knowledge, participation motivation, and participation opportunity) and tourists’ pro-environmental behavior are mediated by environmental self-identity and environmental commitment. These results are similar to the findings of Qasim et al. (2019) and Wang et al. (2019, 2021a, 2021b), whose researches have supported the mediating effect of environmental self-identity and environmental commitment in the relationship between antecedents and many sustainable behaviors. In general, the higher the level of tourists’ environmental knowledge, the more aware they are of environmental issues, and they will have a more positive emotional attitude toward the environment. Consequently, tourists’ affective responses to environmental self-identity and environmental commitment are stronger, resulting in enhanced pro-environmental behavior. Participation motivation actively drives tourists’ environmental self-identity and environmental commitment, which in turn leads to pro-environmental behavior. In addition, participation opportunity suggests that government policies or destination facility support provide specific guidance and a convenient environment for tourists to perform pro-environmental behavior. This influences tourists’ self-judgment and enhances individual pro-environmental behavior by increasing the level of environmental self-identity and environmental commitment.

### Theoretical and managerial implications

#### Theoretical implications

This study makes a theoretical contribution to advancing the research on tourists’ pro-environmental behavior. Based on empirical tests, this research provides new ideas for exploring tourists’ pro-environmental behavior. On one hand, in the SOR theoretical framework, this research introduces the CAC theory and MOA theory to explore the antecedents of tourists’ pro-environmental behavior. Our findings show that environmental knowledge, participation motivation, and opportunity have a positive impact on tourists’ pro-environmental behavior. This research is the first time to integrate the CAC and MOA theories to consider individual pro-environmental behavior in tourism context, which extends the application of related theories in the tourism domain.

On the other hand, to deeply analyze the driving mechanisms of tourists’ pro-environmental behavior, the study incorporates the person-environment relationship into the model framework. Previous studies have mainly explored the possible driving factors of tourist pro-environmental behavior from the perspective of the person-place relationship (Ramkissoon et al., 2013a,b; Lee et al., 2019; Li et al., 2020), while the literature on tourists’ pro-environmental behavior from the perspective of person-environment relationship is limited. This study incorporated two relationship variables (environmental self-identity and environmental commitment) and explored their effects on tourists’ pro-environmental behavior. The findings suggest that the person-environment relationship (environmental self-identity and environmental commitment) plays a significant positive role in enhancing tourists’ pro-environmental behavior. The current research on their relationship with pro-environmental behavior is lacking, while the results in this research provide further support for the influence of environmental self-identity and environmental commitment on tourists’ pro-environmental behavior. The findings highlight the importance of enhancing tourists’ environmental self-identity and environmental commitment in travel and provide insights for understanding the relationship between environmental self-identity, environmental commitment, and pro-environmental behavior.

#### Managerial implications

From a practical perspective, the findings from this work provide some guidance on enhancing tourists’ pro-environmental behavior. The results show that the level of environmental knowledge significantly affects the pro-environmental behavior of tourists. Therefore, the government should strengthen the popularization of environmental knowledge and continuously enhance social awareness of environmental protection. It is necessary to actively seek ways to translate environmental knowledge into real pro-environmental action. It should strengthen the breadth and depth of environmental knowledge popularization through social media platform publicity and organizing expert knowledge lectures. Tourist destination managers can organize plentiful environmental education activities in scenic spots to disseminate scientific knowledge and general knowledge about the environment and protection skills to tourists. As tourists’ environmental knowledge grows, their self-identity and commitment to environmental protection will strengthen, which eventually transform into actual pro-environmental behavior. In the meantime, tourism participation motivation also positively stimulates tourists’ pro-environmental behavior. Destination managers can encourage tourist pro-environmental behavior by setting external incentives. It can also enhance tourism participation motivation in the form of internal incentives by promoting tourists’ environmental concern and environmental responsibility, which will promote tourists’ pro-environmental behavior.

In addition, given the facilitating role of participation opportunity, destinations should provide a convenient environment and material support for tourists to carry out pro-environmental behavior, by continuously improving ecological infrastructure construction and enhancing the supply of pro-environmental tourism products. Government and destination managers should also actively develop management policies to provide specific guidance for regulating tourists’ behavior and enhancing pro-environmental behavior. Finally, in view of the important role of environmental self-identity and environmental commitment, destination managers should actively explore ways to enhance tourists’ environmental self-identity and environmental commitment. Aiming to enhance the awareness and connection of the person-environment relationship, tourism destinations can use multiple social media platforms to widely promote their good ecological environment and continuously create a social atmosphere for eco-environmental protection. These favorable external environments will trigger tourists’ appreciation and approval of the destination, inspire them to develop an environmental identity, make positive environmental commitments, and in turn exhibit specific pro-environmental behavior.

### Limitations and future research directions

The limitations of this work provide insights for further research. First, this study investigated tourists’ subjective environmental knowledge through self-reporting, and the tourists’ environmental knowledge level may be overestimated. Therefore, future researchers need to adopt a more comprehensive approach to examine the tourists’ objective environmental knowledge to more accurately assess the level of environmental knowledge of tourists. Second, many researchers have classified tourism motivation in terms of push-pull motivation, altruistic motivation, and egoistic motivation. Although this research measured tourists’ motivation in terms of both egoistic and altruistic motivation when collecting data, the empirical analysis did not explore whether the two motivations differ in their effects on tourists’ pro-environmental behavior. Hence, we suggest that future research could further explore the mechanism differences in the effects of egoistic and altruistic motivations on tourists’ pro-environmental behavior. Third, future research can further analyze other antecedents, such as social norms, values, risk perceptions, and destination image, in order to fully reveal the driving mechanisms of tourists’ pro-environmental behavior. In some research, scholars have considered the boundary condition that affects tourists’ pro-environmental behavior, such as destination reputation and environmental level (Wang et al., 2019; Su et al., 2020). In future research, the moderating role of destination reputation, environmental level, and service quality should be considered in the research model framework as well. Finally, this research was moderately adjusted to examine Chinese tourists to improve the accuracy of the questionnaire measurement. In future research, we suggest considering validating the findings from different cultural contexts.

## Data availability statement

The raw data supporting the conclusions of this article will be made available by the authors, without undue reservation.

## Ethics statement

Ethical review and approval was not required for the study on human participants in accordance with the local legislation and institutional requirements. The patients/participants provided their written informed consent to participate in this study.

## Author contributions

HT: data collection, literature review, data compiling, data analysis, and text formulation. YM: development of research concept, research framework development and guidance, expert editorial guidance, and funding acquisition. JR: research framework development and guidance, expert editorial guidance and text edition and modification. All authors contributed to the article and approved the submitted version.

## Funding

This research was funded by the Project of National Social Science Fund of China (19BJL036).

## Conflict of interest

The authors declare that the research was conducted in the absence of any commercial or financial relationships that could be construed as a potential conflict of interest.

## Publisher’s note

All claims expressed in this article are solely those of the authors and do not necessarily represent those of their affiliated organizations, or those of the publisher, the editors and the reviewers. Any product that may be evaluated in this article, or claim that may be made by its manufacturer, is not guaranteed or endorsed by the publisher.

## Footnotes

^1^ www.wjx.cn

## References

[ref1] Aas C. Ladkin A. Fletcher J. (2005). Stakeholder collaboration and heritage management. Ann. Tour. Res. 32, 28–48. doi: 10.1016/j.annals.2004.04.005

[ref2] Abdullah S. I. N. W. Samdin Z. Ho J. A. Ng S. I. (2019). Sustainability of marine parks: is knowledge–attitude–behaviour still relevant? Environ. Dev. Sustain. 22, 7357–7384. doi: 10.1007/s10668-019-00524-z

[ref3] Afsar B. Umrani W. A. (2019). Corporate social responsibility and pro-environmental behavior at workplace: the role of moral reflectiveness, coworker advocacy, and environmental commitment. Corp. Soc. Responsib. Environ. Manag. 27, 109–125. doi: 10.1002/csr.1777

[ref4] Agapito D. Oom Do Valle P. Da Costa Mendes J. (2012). The cognitive-affective-conative model of destination image: A confirmatory analysis. J. Travel Tour. Mark. 30, 471–481. doi: 10.1080/10548408.2013.803393

[ref5] Agnew C. R. Lange P. A. M. V. Rusbult C. Langston C. (1998). Cognitive interdependence: commitment and the mental representation of close relationships. J. Pers. Soc. Psychol. 74, 939–954. doi: 10.1037/0022-3514.74.4.939

[ref6] Ahmad A. Thyagaraj K. S. (2015). Consumer's intention to purchase Green brands: the roles of environmental concern, environmental knowledge and self expressive benefits. Curr. World Environ. 10, 879–889. doi: 10.12944/cwe.10.3.18

[ref7] Ahn J. Back K.-J. (2017). Influence of brand relationship on customer attitude toward integrated resort brands: a cognitive, affective, and conative perspective. J. Travel Tour. Mark. 35, 449–460. doi: 10.1080/10548408.2017.1358239

[ref8] Amoah A. Addoah T. (2020). Does environmental knowledge drive pro-environmental behaviour in developing countries? Evidence from households in Ghana. Environ. Dev. Sustain. 23, 2719–2738. doi: 10.1007/s10668-020-00698-x

[ref9] Ateş H. (2020). Merging theory of planned behavior and value identity personal norm model to explain pro-environmental behaviors. Sustain. Prod. Consump. 24, 169–180. doi: 10.1016/j.spc.2020.07.006

[ref10] Attiq S. Rasool H. Iqbal S. (2017). The impact of supportive work environment, trust, and self-efficacy on organizational learning and its effectiveness: A stimulus-organism response approach. Bus. Econ. Rev. 9, 73–100. doi: 10.22547/ber/9.2.4

[ref11] Baird J. Dale G. Holzer J. M. Hutson G. Ives C. D. Plummer R. (2022). The role of a nature-based program in fostering multiple connections to nature. Sustain. Sci. 17, 1899–1910. doi: 10.1007/s11625-022-01119-w

[ref12] Balaji M. S. Jiang Y. Jha S. (2019). Green hotel adoption: a personal choice or social pressure? Int. J. Contemp. Hosp. Manag. 31, 3287–3305. doi: 10.1108/ijchm-09-2018-0742

[ref13] Balunde A. Perlaviciute G. Steg L. (2019). The relationship between People's environmental considerations and pro-environmental behavior in Lithuania. Front. Psychol. 10:2319. doi: 10.3389/fpsyg.2019.02319, PMID: 31681111 PMC6803424

[ref14] Benner M. (2020). Overcoming overtourism in Europe: towards an institutional-behavioral research agenda. Zeitschrift für Wirtschaftsgeographie 64, 74–87. doi: 10.1515/zfw-2019-0016

[ref15] Betta L. Dattilo B. di Bella E. Finocchiaro G. Iaccarino S. (2021). Tourism and road transport emissions in Italy. Sustainability 13:12712. doi: 10.3390/su132212712

[ref16] Bord R. J. O'Connor R. E. Fisher A. (2016). In what sense does the public need to understand global climate change? Public Underst. Sci. 9, 205–218. doi: 10.1088/0963-6625/9/3/301

[ref17] Brügger A. Kaiser F. G. Kaiser F. G. Roczen N. Roczen N. Roczen N. (2011). One for all? Connectedness to nature, inclusion of nature, environmental identity, and implicit association with nature. Eur. Psychol. 16, 324–333. doi: 10.1027/1016-9040/a000032

[ref18] Cantor D. E. Morrow P. C. Montabon F. (2012). Engagement in environmental behaviors among supply chain management employees: an organizational support theoretical perspective. J. Supply Chain Manag. 48, 33–51. doi: 10.1111/j.1745-493X.2011.03257.x

[ref19] Carfora V. Cavallo C. Caso D. Del Giudice T. De Devitiis B. Viscecchia R.. (2019). Explaining consumer purchase behavior for organic milk: including trust and green self-identity within the theory of planned behavior. Food Qual. Prefer. 76, 1–9. doi: 10.1016/j.foodqual.2019.03.006

[ref20] Chen M. F. (2020). The impacts of perceived moral obligation and sustainability self-identity on sustainability development: A theory of planned behavior purchase intention model of sustainability-labeled coffee and the moderating effect of climate change skepticism. Bus. Strateg. Environ. 29, 2404–2417. doi: 10.1002/bse.2510

[ref21] Chen G. So K. K. F. Hu X. Poomchaisuwan M. (2021). Travel for affection: A stimulus-organism-response model of honeymoon tourism experiences. J. Hosp. Tour. Res. 46, 1187–1219. doi: 10.1177/10963480211011720

[ref22] Cheng T.-M. Wu H. C. (2014). How do environmental knowledge, environmental sensitivity, and place attachment affect environmentally responsible behavior? An integrated approach for sustainable island tourism. J. Sustain. Tour. 23, 557–576. doi: 10.1080/09669582.2014.965177

[ref23] Chiu Y.-T. H. Lee W.-I. Chen T.-H. (2014). Environmentally responsible behavior in ecotourism: antecedents and implications. Tour. Manag. 40, 321–329. doi: 10.1016/j.tourman.2013.06.013

[ref24] Chwialkowska A. Bhatti W. A. Glowik M. (2020). The influence of cultural values on pro-environmental behavior. J. Clean. Prod. 268:122305. doi: 10.1016/j.jclepro.2020.122305

[ref25] Clark C. F. Kotchen M. J. Moore M. R. (2003). Internal and external influences on pro-environmental behavior: participation in a green electricity program. J. Environ. Psychol. 23, 237–246. doi: 10.1016/s0272-4944(02)00105-6

[ref26] Cohen A. (2007). Commitment before and after: an evaluation and reconceptualization of organizational commitment. Hum. Resour. Manag. Rev. 17, 336–354. doi: 10.1016/j.hrmr.2007.05.001

[ref27] Cook A. Kerr G. N. Moore K. (2002). Attitudes and intentions towards purchasing GM food. J. Econ. Psychol. 23, 557–572. doi: 10.1016/S0167-4870(02)00117-4

[ref28] Danish A. Wang Z. (2019). Dynamic relationship between tourism, economic growth, and environmental quality. J. Sustain. Tour. 26, 1928–1943. doi: 10.1080/09669582.2018.1526293

[ref29] Davis J. L. Green J. D. Reed A. (2009). Interdependence with the environment: commitment, interconnectedness, and environmental behavior. J. Environ. Psychol. 29, 173–180. doi: 10.1016/j.jenvp.2008.11.001

[ref30] Davis J. L. Le B. Coy A. E. (2011). Building a model of commitment to the natural environment to predict ecological behavior and willingness to sacrifice. J. Environ. Psychol. 31, 257–265. doi: 10.1016/j.jenvp.2011.01.004

[ref31] Dermody J. Hanmer-Lloyd S. Koenig-Lewis N. Zhao A. L. (2015). Advancing sustainable consumption in the UK and China: the mediating effect of pro-environmental self-identity. J. Mark. Manag. 31, 1472–1502. doi: 10.1080/0267257x.2015.1061039

[ref32] Emmerich P. Hülemeier A.-G. Jendryczko D. Baumann M. J. Weil M. Baur D. (2020). Public acceptance of emerging energy technologies in context of the German energy transition. Energy Policy 142:111516. doi: 10.1016/j.enpol.2020.111516

[ref33] Fanghella V. d'Adda G. Tavoni M. (2019). On the use of nudges to affect spillovers in environmental behaviors. Front. Psychol. 10:61. doi: 10.3389/fpsyg.2019.00061, PMID: 30761038 PMC6362870

[ref34] Faraz N. A. Ahmed F. Ying M. Mehmood S. A. (2021). The interplay of green servant leadership, self-efficacy, and intrinsic motivation in predicting employees' pro-environmental behavior. Corp. Soc. Responsib. Environ. Manag. 28, 1171–1184. doi: 10.1002/csr.2115

[ref35] Fenitra R. M. Tanti H. Gancar C. P. Indrianawati U. Hartini S. (2021). Extended theory of planned behavior to explain Environemntally responsible behavior in context of nature-based tourism. Geo J. Tour. Geosites 39, 1507–1516. doi: 10.30892/gtg.394spl22-795

[ref36] Fornell C. Larcker D. F. (1981). Evaluating structural equation models with unobservable variables and measurement error. J. Mark. Res. 18, 39–50. doi: 10.1177/002224378101800104

[ref37] Fryxell G. E. Lo C. W. H. (2003). The influence of environmental knowledge and values on managerial Behaviours on behalf of the environment: an empirical examination of managers in China. J. Bus. Ethics 46, 45–69. doi: 10.1023/A:1024773012398

[ref38] Geng J. Long R. Chen H. Yue T. Li W. Li Q. (2017). Exploring multiple motivations on urban Residents' travel mode choices: an empirical Study from Jiangsu Province in China. Sustainability 9:136. doi: 10.3390/su9010136

[ref39] Gil-Giménez D. Rolo-González G. Suárez E. Muinos G. (2021). The influence of environmental self-identity on the relationship between consumer identities and frugal behavior. Sustainability 13:9664. doi: 10.3390/su13179664

[ref40] González-Rodríguez M. R. Díaz-Fernández M. C. (2020). Customers' corporate social responsibility awareness as antecedent of repeat behaviour intention. Corp. Soc. Responsib. Environ. Manag. 27, 1294–1306. doi: 10.1002/csr.1884

[ref41] Graci S. Dodds R. (2008). Why go Green? The business case for environmental commitment in the Canadian hotel industry. Anatolia 19, 251–270. doi: 10.1080/13032917.2008.9687072

[ref42] Hair J. F. Risher J. J. Sarstedt M. Ringle C. M. (2019). When to use and how to report the results of PLS-SEM. Eur. Bus. Rev. 31, 2–24. doi: 10.1108/ebr-11-2018-0203

[ref43] Hair J. Sarstedt M. Hopkins L. Kuppelwieser V. (2014). Partial least squares structural equation modeling (PLS-SEM). Eur. Bus. Rev. 26, 106–121. doi: 10.1108/ebr-10-2013-0128

[ref44] Halpenny E. A. (2010). Pro-environmental behaviours and park visitors: the effect of place attachment. J. Environ. Psychol. 30, 409–421. doi: 10.1016/j.jenvp.2010.04.006

[ref45] Han H. (2015). Travelers' pro-environmental behavior in a green lodging context: converging value-belief-norm theory and the theory of planned behavior. Tour. Manag. 47, 164–177. doi: 10.1016/j.tourman.2014.09.014

[ref46] Han H. (2021). Consumer behavior and environmental sustainability in tourism and hospitality: a review of theories, concepts, and latest research. J. Sustain. Tour. 29, 1021–1042. doi: 10.1080/09669582.2021.1903019

[ref47] Han W. McCabe S. Wang Y. Chong A. Y. L. (2017). Evaluating user-generated content in social media: an effective approach to encourage greater pro-environmental behavior in tourism? J. Sustain. Tour. 26, 600–614. doi: 10.1080/09669582.2017.1372442

[ref48] Hayati Y. Adrianto L. Krisanti M. Pranowo W. S. Kurniawan F. (2020). Magnitudes and tourist perception of marine debris on small tourism island: assessment of Tidung Island, Jakarta, Indonesia. Mar Pollut Bull 158:111393. doi: 10.1016/j.marpolbul.2020.111393, PMID: 32753179

[ref49] He X. Cheng J. Swanson S. R. Su L. Hu D. (2022). The effect of destination employee service quality on tourist environmentally responsible behavior: A moderated mediation model incorporating environmental commitment, destination social responsibility and motive attributions. Tour. Manag. 90:104470. doi: 10.1016/j.tourman.2021.104470

[ref50] He X. Hu D. Swanson S. R. Su L. Chen X. (2018). Destination perceptions, relationship quality, and tourist environmentally responsible behavior. Tour. Manag. Perspect. 28, 93–104. doi: 10.1016/j.tmp.2018.08.001

[ref51] Henseler J. Ringle C. M. Sarstedt M. (2014). A new criterion for assessing discriminant validity in variance-based structural equation modeling. J. Acad. Mark. Sci. 43, 115–135. doi: 10.1007/s11747-014-0403-8

[ref52] Hergesell A. (2017). Environmental commitment in holiday transport mode choice. Int. J. Cult. Tour. Hosp. Res. 11, 67–80. doi: 10.1108/ijcthr-09-2015-0118

[ref53] Hilgard E. R. (1980). The trilogy of mind: cognition, affection, and conation. J. Hist. Behav. Sci. 16, 107–117. doi: 10.1002/1520-6696(198004)16:2<107::AID-JHBS2300160202>3.0.CO;2-Y, PMID: 11608381

[ref54] Hirunyawipada T. Xiong G. (2018). Corporate environmental commitment and financial performance: moderating effects of marketing and operations capabilities. J. Bus. Res. 86, 22–31. doi: 10.1016/j.jbusres.2018.01.002

[ref55] Hsiao C.-C. (2020). Understanding content sharing on the internet: test of a cognitive-affective-conative model. Online Inf. Rev. 44, 1289–1306. doi: 10.1108/oir-11-2019-0350

[ref56] Hu L. T. Bentler P. M. (1999). Cutoff criteria for fit indexes in covariance structure analysis: conventional criteria versus new alternatives. Struct. Equ. Model. 6, 1–55. doi: 10.1080/10705519909540118

[ref57] Hu H. Zhang J. Chu G. Yang J. Yu P. (2018). Factors influencing tourists' litter management behavior in mountainous tourism areas in China. Waste Manag. 79, 273–286. doi: 10.1016/j.wasman.2018.07.047, PMID: 30343755

[ref58] Huang Y.-M. Lou S.-J. Huang T.-C. Jeng Y.-L. (2018). Middle-aged adults' attitudes toward health app usage: a comparison with the cognitive-affective-conative model. Univ. Access Inf. Soc. 18, 927–938. doi: 10.1007/s10209-018-0621-9

[ref59] Huber J. Viscusi W. K. Bell J. (2018). Dynamic relationships between social norms and pro-environmental behavior: evidence from household recycling. Behav. Public Policy 4, 1–25. doi: 10.1017/bpp.2017.13

[ref60] Ienna M. Rofe A. Gendi M. Douglas H. E. Kelly M. Hayward M. W.. (2022). The relative role of knowledge and empathy in predicting pro-environmental attitudes and behavior. Sustainability 14:4622. doi: 10.3390/su14084622

[ref61] Iftikhar U. Zaman K. Rehmani M. Ghias W. Islam T. (2021). Impact of Green human resource management on service recovery: mediating role of environmental commitment and moderation of transformational leadership. Front. Psychol. 12:710050. doi: 10.3389/fpsyg.2021.710050, PMID: 34759860 PMC8574843

[ref62] Jiang J. (2020). The role of natural soundscape in nature-based tourism experience: an extension of the stimulus–organism–response model. Curr. Issue Tour. 25, 707–726. doi: 10.1080/13683500.2020.1859995

[ref63] Juvan E. Dolnicar S. (2017). Drivers of pro-environmental tourist behaviours are not universal. J. Clean. Prod. 166, 879–890. doi: 10.1016/j.jclepro.2017.08.087

[ref64] Kamboj S. Sarmah B. Gupta S. Dwivedi Y. (2018). Examining branding co-creation in brand communities on social media: applying the paradigm of stimulus-organism-response. Int. J. Inf. Manag. 39, 169–185. doi: 10.1016/j.ijinfomgt.2017.12.001

[ref65] Kellner A. Cafferkey K. Townsend K. (2019). “Ability, motivation and opportunity theory: a formula for employee performance?” in Elgar Introduction to Theories of Human Resources and Employment Relations. eds. K. Townsend, K. Cafferkey, A. M. McDermott, and T. Dundon (Cheltenham, Edward Elgar), 311–323.

[ref66] Kiatkawsin K. Han H. (2017). Young travelers' intention to behave pro-environmentally: merging the value-belief-norm theory and the expectancy theory. Tour. Manag. 59, 76–88. doi: 10.1016/j.tourman.2016.06.018

[ref67] Kim Y. H. Kim D. J. Wachter K. (2013). A study of mobile user engagement (MoEN): engagement motivations, perceived value, satisfaction, and continued engagement intention. Decis. Support. Syst. 56, 361–370. doi: 10.1016/j.dss.2013.07.002

[ref68] Kim M. J. Lee C.-K. Jung T. (2018). Exploring consumer behavior in virtual reality tourism using an extended stimulus-organism-response model. J. Travel Res. 59, 69–89. doi: 10.1177/0047287518818915

[ref69] Kim M.-S. Stepchenkova S. (2019). Altruistic values and environmental knowledge as triggers of pro-environmental behavior among tourists. Curr. Issue Tour. 23, 1575–1580. doi: 10.1080/13683500.2019.1628188

[ref70] Klein H. J. Cooper J. T. Molloy J. C. Swanson J. A. (2014). The assessment of commitment: advantages of a unidimensional, target-free approach. J. Appl. Psychol. 99, 222–238. doi: 10.1037/a0034751, PMID: 24188389

[ref71] Kollmuss A. Agyeman J. (2010). Mind the gap: why do people act environmentally and what are the barriers to pro-environmental behavior? Environ. Educ. Res. 8, 239–260. doi: 10.1080/13504620220145401

[ref72] Kwon J. Boger C. A. (2020). Influence of brand experience on customer inspiration and pro-environmental intention. Curr. Issue Tour. 24, 1154–1168. doi: 10.1080/13683500.2020.1769571

[ref73] Landon A. C. Woosnam K. M. Boley B. B. (2018). Modeling the psychological antecedents to tourists' pro-sustainable behaviors: an application of the value-belief-norm model. J. Sustain. Tour. 26, 957–972. doi: 10.1080/09669582.2017.1423320

[ref74] Lavidge R. J. Steiner G. A. (1961). A model for predictive measurements of advertising effectiveness. J. Mark. 25, 59–62. doi: 10.1177/002224296102500611

[ref75] Lee S. Ha S. Widdows R. (2011). Consumer responses to high-technology products: product attributes, cognition, and emotions. J. Bus. Res. 64, 1195–1200. doi: 10.1016/j.jbusres.2011.06.022

[ref76] Lee T. H. Jan F.-H. Yang C.-C. (2013). Conceptualizing and measuring environmentally responsible behaviors from the perspective of community-based tourists. Tour. Manag. 36, 454–468. doi: 10.1016/j.tourman.2012.09.012

[ref77] Lee Y.-K. Pei F. Ryu K. Choi S. (2019). Why the tripartite relationship of place attachment, loyalty, and pro-environmental behaviour matter? Asia Pac. J. Tour. Res. 24, 250–267. doi: 10.1080/10941665.2018.1564344

[ref78] Lepoša N. (2017). The emergence of ambivalent leisure consumers – the case of boating along the Swedish west coast. J. Clean. Prod. 145, 35–44. doi: 10.1016/j.jclepro.2017.01.002

[ref79] Leung X. Y. Bai B. (2013). How motivation, opportunity, and ability impact Travelers' social media involvement and revisit intention. J. Travel Tour. Mark. 30, 58–77. doi: 10.1080/10548408.2013.751211

[ref80] Li Q. Li X. Chen W. C. Su X. Yu R. (2020). Involvement, place attachment, and environmentally responsible behaviour connected with geographical indication products. Tour. Geogr., 1–26. doi: 10.1080/14616688.2020.1826569

[ref81] Li D. Xu X. Chen C.-F. Menassa C. (2019a). Understanding energy-saving behaviors in the American workplace: A unified theory of motivation, opportunity, and ability. Energy Res. Soc. Sci. 51, 198–209. doi: 10.1016/j.erss.2019.01.020

[ref82] Li D. Zhao L. Ma S. Shao S. Zhang L. (2019b). What influences an individual's pro-environmental behavior? A literature review. Resour. Conserv. Recycl. 146, 28–34. doi: 10.1016/j.resconrec.2019.03.024

[ref83] Lim S. H. Kim D. J. (2020). Does emotional intelligence of online shoppers affect their shopping behavior? From a cognitive-affective-conative framework perspective. Int. J. Hum. Comput. Interaction 36, 1304–1313. doi: 10.1080/10447318.2020.1739882

[ref84] Lin Z. (2022). The impact of marine tourism behavior on the ecological effect of marine benthos Community in the South China Sea. J. Coast. Conserv. 26:17. doi: 10.1007/s11852-022-00864-5

[ref85] Lin W. Li Y. Li X. Xu D. (2018). The dynamic analysis and evaluation on tourist ecological footprint of city: take Shanghai as an instance. Sustain. Cities Soc. 37, 541–549. doi: 10.1016/j.scs.2017.12.003

[ref86] Liobikienė G. Poškus M. S. (2019). The importance of environmental knowledge for private and public sphere pro-environmental behavior: modifying the value-belief-norm theory. Sustainability 11:3324. doi: 10.3390/su11123324

[ref87] Liu J. Li J. Jang S. Zhao Y. (2022). Understanding tourists' environmentally responsible behavior at coastal tourism destinations. Mar. Policy 143:105178. doi: 10.1016/j.marpol.2022.105178

[ref88] Liu Z. Li J. Zhu H. Cai Z. Wang L. (2012). Chinese firms' sustainable development—the role of future orientation, environmental commitment, and employee training. Asia Pac. J. Manag. 31, 195–213. doi: 10.1007/s10490-012-9291-y

[ref89] Liu L. Shi X. Q. (2021). Research on the impact mechanism of sports tourism consumption behavior under the background ofthe normalization of COVID-19 prevention and control:empirical analysis of MOA-TAM integration model based on S-O-R framework. Tour. Tribune 36, 52–70. doi: 10.19765/j.cnki.1002-5006.2021.08.010. (in Chinese)

[ref90] Liu P. Teng M. Han C. (2020). How does environmental knowledge translate into pro-environmental behaviors?: the mediating role of environmental attitudes and behavioral intentions. Sci. Total Environ. 728:138126. doi: 10.1016/j.scitotenv.2020.138126, PMID: 32361356

[ref91] Liu J. Wu J. S. Che T. (2019). Understanding perceived environment quality in affecting tourists' environmentally responsible behaviours: A broken windows theory perspective. Tour. Manag. Perspect. 31, 236–244. doi: 10.1016/j.tmp.2019.05.007

[ref92] Lorenzo-Romero C. Alarcon-Del-Amo M. D. Gomez-Borja M. A. (2016). Analyzing the user behavior toward electronic commerce stimuli. Front. Behav. Neurosci. 10:224. doi: 10.3389/fnbeh.2016.00224, PMID: 27965549 PMC5127800

[ref93] Luo W. Tang P. Jiang L. Su M. M. (2020). Influencing mechanism of tourist social responsibility awareness on environmentally responsible behavior. J. Clean. Prod. 271:122565. doi: 10.1016/j.jclepro.2020.122565

[ref94] MacInnis D. J. Jaworski B. J. (1989). Information processing from advertisements: toward an integrative framework. J. Mark. 53, 1–23. doi: 10.1177/002224298905300401

[ref95] Manthiou A. Ayadi K. Lee S. Chiang L. Tang L. (2016). Exploring the roles of self-concept and future memory at consumer events: the application of an extended Mehrabian–Russell model. J. Travel Tour. Mark. 34, 531–543. doi: 10.1080/10548408.2016.1208786

[ref96] Masterson V. A. Stedman R. C. Enqvist J. Tengö M. Giusti M. Wahl D.. (2017). The contribution of sense of place to social-ecological systems research: a review and research agenda. Ecol. Soc. 22:49. doi: 10.5751/es-08872-220149

[ref97] Mehrabian A. Russell J. A. (1974). An Approach to Environmental Psychology, Cambridge: The MIT Press.

[ref98] Meyer J. P. Allen N. J. (1991). A three-component conceptualization of organizational commitment. Hum. Resour. Manag. Rev. 1, 61–89. doi: 10.1016/1053-4822(91)90011-Z

[ref99] Meyer J. P. Herscovitch L. (2001). Commitment in the workplace: toward a general model. Hum. Resour. Manag. Rev. 11, 299–326. doi: 10.1016/S1053-4822(00)00053-X

[ref100] Mostafa M. M. (2007). Gender differences in Egyptian consumers? Green purchase behaviour: the effects of environmental knowledge, concern and attitude. Int. J. Consum. Stud. 31, 220–229. doi: 10.1111/j.1470-6431.2006.00523.x

[ref101] Oliver R. L. (1999). Whence consumer loyalty. J. Mark. 63, 33–44. doi: 10.1177/00222429990634s105

[ref102] Onel N. Mukherjee A. (2016). Consumer knowledge in pro-environmental behavior. World J. Sci. Technol. Sustain. Dev. 13, 328–352. doi: 10.1108/wjstsd-01-2016-0004

[ref103] Paco A. Lavrador T. (2017). Environmental knowledge and attitudes and behaviours towards energy consumption. J. Environ. Manag. 197, 384–392. doi: 10.1016/j.jenvman.2017.03.100, PMID: 28410516

[ref104] Park J. Stoel L. Lennon S. J. (2008). Cognitive, affective and conative responses to visual simulation: the effects of rotation in online product presentation. J. Consum. Behav. 7, 72–87. doi: 10.1002/cb.237

[ref105] Pham N. T. Tučková Z. Chiappetta Jabbour C. J. (2019). Greening the hospitality industry: how do green human resource management practices influence organizational citizenship behavior in hotels? A mixed-methods study. Tour. Manag. 72, 386–399. doi: 10.1016/j.tourman.2018.12.008

[ref106] Podsakoff P. M. MacKenzie S. B. Lee J.-Y. Podsakoff N. P. (2003). Common method biases in behavioral research: a critical review of the literature and recommended remedies. J. Appl. Psychol. 88, 879–903. doi: 10.1037/0021-9010.88.5.879, PMID: 14516251

[ref107] Preziosi M. Tourais P. Acampora A. Videira N. Merli R. (2019). The role of environmental practices and communication on guest loyalty: examining EU-ecolabel in Portuguese hotels. J. Clean. Prod. 237:117659. doi: 10.1016/j.jclepro.2019.117659

[ref108] Qasim H. Yan L. Guo R. Saeed A. Ashraf B. N. (2019). The defining role of environmental self-identity among consumption values and behavioral intention to consume organic food. Int. J. Environ. Res. Public Health 16:1106. doi: 10.3390/ijerph16071106, PMID: 30925666 PMC6479335

[ref109] Rahman I. Reynolds D. (2016). Predicting green hotel behavioral intentions using a theory of environmental commitment and sacrifice for the environment. Int. J. Hosp. Manag. 52, 107–116. doi: 10.1016/j.ijhm.2015.09.007

[ref110] Raineri N. Paillé P. (2015). Linking corporate policy and supervisory support with environmental citizenship behaviors: the role of employee environmental beliefs and commitment. J. Bus. Ethics 137, 129–148. doi: 10.1007/s10551-015-2548-x

[ref111] Ramkissoon H. Graham Smith L. D. Weiler B. (2013a). Testing the dimensionality of place attachment and its relationships with place satisfaction and pro-environmental behaviours: A structural equation modelling approach. Tour. Manag. 36, 552–566. doi: 10.1016/j.tourman.2012.09.003

[ref112] Ramkissoon H. Smith L. D. G. Weiler B. (2013b). Relationships between place attachment, place satisfaction and pro-environmental behaviour in an Australian national park. J. Sustain. Tour. 21, 434–457. doi: 10.1080/09669582.2012.708042

[ref113] Ramkissoon H. Weiler B. Smith L. D. G. (2012). Place attachment and pro-environmental behaviour in national parks: the development of a conceptual framework. J. Sustain. Tour. 20, 257–276. doi: 10.1080/09669582.2011.602194

[ref114] Rose S. Clark M. Samouel P. Hair N. (2012). Online customer experience in e-retailing: an empirical model of antecedents and outcomes. J. Retail. 88, 308–322. doi: 10.1016/j.jretai.2012.03.001

[ref115] Saari U. A. Damberg S. Frömbling L. Ringle C. M. (2021). Sustainable consumption behavior of Europeans: the influence of environmental knowledge and risk perception on environmental concern and behavioral intention. Ecol. Econ. 189:107155. doi: 10.1016/j.ecolecon.2021.107155

[ref116] Safari A. Salehzadeh R. Panahi R. Abolghasemian S. (2018). Multiple pathways linking environmental knowledge and awareness to employees' green behavior. Corpor. Gov. Int. J. Bus. Soc. 18, 81–103. doi: 10.1108/cg-08-2016-0168

[ref117] Sahabuddin M. Tan Q. Hossain I. Alam M. S. Nekmahmud M. (2021). Tourist environmentally responsible behavior and satisfaction; study on the World's longest Natural Sea beach, Cox's Bazar, Bangladesh. Sustainability 13:9383. doi: 10.3390/su13169383

[ref118] Schlittgen R. Ringle C. M. Sarstedt M. Becker J.-M. (2015). Segmentation of PLS path models by iterative reweighted regressions. J. Bus. Res. 69, 4583–4592. doi: 10.1016/j.jbusres.2016.04.009

[ref119] Sibian A.-R. Ispas A. (2021). An approach to applying the ability-motivation-opportunity theory to identify the driving factors of Green employee behavior in the hotel industry. Sustainability 13:4659. doi: 10.3390/su13094659

[ref120] Somjai S. Fongtanakit R. Laosillapacharoen K. (2020). Impact of environmental commitment, environmental management accounting and Green innovation on firm performance: an empirical investigation. Int. J. Energy Econ. Policy 10, 204–210. doi: 10.32479/ijeep.9174

[ref121] Stern P. C. Dietz T. Abel T. D. Guagnano G. A. Kalof L. (1999). A value-belief-norm theory of support for social movements: the case of environmentalism. Hum. Ecol. Rev. 6, 81–97.

[ref122] Su L. Huang S. Pearce J. (2019). Toward a model of destination resident–environment relationship: the case of Gulangyu, China. J. Travel Tour. Mark. 36, 469–483. doi: 10.1080/10548408.2019.1568954

[ref123] Su L. Lian Q. Huang Y. (2020). How do tourists' attribution of destination social responsibility motives impact trust and intention to visit? The moderating role of destination reputation. Tour. Manag. 77:103970. doi: 10.1016/j.tourman.2019.103970

[ref124] Su L. Swanson S. R. (2017). The effect of destination social responsibility on tourist environmentally responsible behavior: compared analysis of first-time and repeat tourists. Tour. Manag. 60, 308–321. doi: 10.1016/j.tourman.2016.12.011

[ref125] Tabernero C. Hernandez B. (2012). A motivational model for environmentally responsible behavior. Span. J. Psychol. 15, 648–658. doi: 10.5209/rev_sjop.2012.v15.n2.38876, PMID: 22774439

[ref126] Taha S. Osaili T. M. Vij A. Albloush A. Nassoura A. (2020). Structural modelling of relationships between food safety knowledge, attitude, commitment and behavior of food handlers in restaurants in Jebel Ali free zone, Dubai, UAE. Food Control 118:107431. doi: 10.1016/j.foodcont.2020.107431

[ref127] Talwar S. Jabeen F. Tandon A. Sakashita M. Dhir A. (2021). What drives willingness to purchase and stated buying behavior toward organic food? A stimulus–organism–behavior–consequence (SOBC) perspective. J. Clean. Prod. 293:125882. doi: 10.1016/j.jclepro.2021.125882

[ref128] Tang Z. Warkentin M. Wu L. (2019). Understanding employees' energy saving behavior from the perspective of stimulus-organism-responses. Resour. Conserv. Recycl. 140, 216–223. doi: 10.1016/j.resconrec.2018.09.030

[ref129] Tian Q. Robertson J. L. (2017). How and when does perceived CSR affect Employees' engagement in voluntary pro-environmental behavior? J. Bus. Ethics 155, 399–412. doi: 10.1007/s10551-017-3497-3

[ref130] Trudel R. Argo J. J. Meng M. D. (2016). The recycled self: Consumers' disposal decisions of identity-linked products. J. Consum. Res. 43, 246–264. doi: 10.1093/jcr/ucw014

[ref131] Ul Islam J. Rahman Z. (2017). The impact of online brand community characteristics on customer engagement: an application of stimulus-organism-response paradigm. Telematics Inform. 34, 96–109. doi: 10.1016/j.tele.2017.01.004

[ref132] Unal A. B. Steg L. Gorsira M. (2018). Values versus environmental knowledge as triggers of a process of activation of personal norms for eco-driving. Environ. Behav. 50, 1092–1118. doi: 10.1177/0013916517728991, PMID: 30473587 PMC6207993

[ref133] Unger R. Abegg B. Mailer M. Stampfl P. (2016). Energy consumption and greenhouse gas emissions resulting from tourism travel in an alpine setting. Mt. Res. Dev. 36, 475–483. doi: 10.1659/mrd-journal-d-16-00058.1

[ref134] Van der Werff E. Steg L. Keizer K. (2013a). I am what I am, by looking past the present. Environ. Behav. 46, 626–657. doi: 10.1177/0013916512475209

[ref135] van der Werff E. Steg L. Keizer K. (2013b). The value of environmental self-identity: the relationship between biospheric values, environmental self-identity and environmental preferences, intentions and behaviour. J. Environ. Psychol. 34, 55–63. doi: 10.1016/j.jenvp.2012.12.006

[ref136] van der Werff E. Steg L. Keizer K. (2014). Follow the signal: when past pro-environmental actions signal who you are. J. Environ. Psychol. 40, 273–282. doi: 10.1016/j.jenvp.2014.07.004

[ref137] van der Werff E. Steg L. Ruepert A. (2021). My company is green, so am I: the relationship between perceived environmental responsibility of organisations and government, environmental self-identity, and pro-environmental behaviours. Energ. Effic. 14:50. doi: 10.1007/s12053-021-09958-9

[ref138] Van Waeyenberg T. Decramer A. (2018). Line managers' AMO to manage employees' performance: the route to effective and satisfying performance management. Int. J. Hum. Resour. Manag. 29, 3093–3114. doi: 10.1080/09585192.2018.1445656

[ref139] Vicente-Molina M. A. Fernández-Sáinz A. Izagirre-Olaizola J. (2013). Environmental knowledge and other variables affecting pro-environmental behaviour: comparison of university students from emerging and advanced countries. J. Clean. Prod. 61, 130–138. doi: 10.1016/j.jclepro.2013.05.015

[ref140] Wang X. B. Chen C. C. Ku G. C. M. Chen C. H. Hsu C. H. Lee P. Y. (2022). Travel for survive! Identifying the antecedents of vaccine tourists' travel intention: using a stimulus-organism-response model. Front. Public Health 10:850154. doi: 10.3389/fpubh.2022.850154, PMID: 36033750 PMC9407439

[ref141] Wang X. Van der Werff E. Bouman T. Harder M. K. Steg L. (2021b). I am vs. we are: how Biospheric values and environmental identity of individuals and groups can influence pro-environmental behaviour. Front. Psychol. 12:618956. doi: 10.3389/fpsyg.2021.618956, PMID: 33679533 PMC7930912

[ref142] Wang S. Wang J. Li J. Yang F. (2019). Do motivations contribute to local residents' engagement in pro-environmental behaviors? Resident-destination relationship and pro-environmental climate perspective. J. Sustain. Tour. 28, 834–852. doi: 10.1080/09669582.2019.1707215

[ref143] Wang S. Wang J. Wang Y. Yan J. Li J. (2018). Environmental knowledge and consumers' intentions to visit green hotels: the mediating role of consumption values. J. Travel Tour. Mark. 35, 1261–1271. doi: 10.1080/10548408.2018.1490234

[ref144] Wang J. Wang S. Wang H. Zhang Z. Ru X. (2021a). Examining when and how perceived sustainability-related climate influences pro-environmental behaviors of tourism destination residents in China. J. Hosp. Tour. Manag. 48, 357–367. doi: 10.1016/j.jhtm.2021.07.008

[ref145] Werff E. V. D. Steg L. Keizer K. (2013). It is a moral issue: the relationship between environmental self-identity, obligation-based intrinsic motivation and pro-environmental behaviour. Glob. Environ. Change Hum. Policy Dimensions 23, 1258–1265. doi: 10.1016/j.gloenvcha.2013.07.018

[ref146] Whitmarsh L. O'Neill S. (2010). Green identity, green living? The role of pro-environmental self-identity in determining consistency across diverse pro-environmental behaviours. J. Environ. Psychol. 30, 305–314. doi: 10.1016/j.jenvp.2010.01.003

[ref147] Wu Z. Geng L. (2020). Traveling in haze: how air pollution inhibits tourists' pro-environmental behavioral intentions. Sci. Total Environ. 707:135569. doi: 10.1016/j.scitotenv.2019.135569, PMID: 31776002

[ref148] Ye Q. Anwar M. A. Zhou R. Asmi F. Ahmad I. (2020). Short stay, long impact: ecological footprints of sojourners. Environ. Sci. Pollut. Res. Int. 27, 11797–11808. doi: 10.1007/s11356-020-07700-z, PMID: 31970643

[ref149] Yoon A. Jeong D. Chon J. (2021). The impact of the risk perception of ocean microplastics on tourists' pro-environmental behavior intention. Sci. Total Environ. 774:144782. doi: 10.1016/j.scitotenv.2020.144782

[ref150] Yusliza M. Y. Amirudin A. Rahadi R. A. Nik Sarah Athirah N. A. Ramayah T. Muhammad Z.. (2020). An investigation of pro-environmental behaviour and sustainable development in Malaysia. Sustainability 12:7083. doi: 10.3390/su12177083

[ref151] Zebardast L. Radaei M. (2022). The influence of global crises on reshaping pro-environmental behavior, case study: the COVID-19 pandemic. Sci. Total Environ. 811:151436. doi: 10.1016/j.scitotenv.2021.151436, PMID: 34742989 PMC8596762

[ref152] Zhang J. Xie C. Morrison A. M. Zhang K. (2020). Fostering resident pro-environmental behavior: the roles of destination image and Confucian culture. Sustainability 12:597. doi: 10.3390/su12020597

[ref153] Zhang H. Zhang Y. Song Z. Lew A. A. (2019). Assessment bias of environmental quality (AEQ), consideration of future consequences (CFC), and environmentally responsible behavior (ERB) in tourism. J. Sustain. Tour. 27, 609–628. doi: 10.1080/09669582.2019.1597102

[ref154] Zhang Y. Zhang H.-L. Zhang J. Cheng S.-W. (2014). Predicting residents' pro-environmental behaviors at tourist sites: the role of awareness of disaster's consequences, values, and place attachment. J. Environ. Psychol. 40, 131–146. doi: 10.1016/j.jenvp.2014.06.001

[ref155] Zhu P. Chi X. Ryu H. B. Ariza-Montes A. Han H. (2022). Traveler pro-social behaviors at heritage tourism sites. Front. Psychol. 10:901530. doi: 10.3389/fpsyg.2022.901530 PMC9226447 35756300

